# An extended inequality approach for evaluating decision making units with a single output

**DOI:** 10.1186/s13660-017-1459-z

**Published:** 2017-08-29

**Authors:** Xiao-Li Meng, Fu-Gui Shi

**Affiliations:** 1 School of Mathematics and Statistics, Beijing Institute of Technology, Beijing, 100081 China; 2 Beijing Key Laboratory on MCAACI, Beijing Institute of Technology, Beijing, 100081 China

**Keywords:** data envelopment analysis, decision making unit, inequality, partially ordered set, minimal element

## Abstract

In this work, an extended evaluation approach for decision making units (DMUs) with a single output is proposed. Firstly, the input and output data for each DMU are changed in the same proportion until all the outputs are equal, and then the coordinate system is established with input *i* as the *i*th coordinate axis. Secondly, in the coordinate system, the production possibility set, which is spanned by all the DMUs without the evaluated DMU, is expressed by inequalities. Moreover, the mathematical expression of the line segment joining the origin to the evaluated DMU is given. Thirdly, the efficiency measure of the evaluated DMU is obtained from the relationship between the production possibility set and the line segment. In order to distinguish the weak efficiency and efficiency, the partially ordered set and minimal element are introduced in the paper. Finally, an example is provided to illustrate the proposed approach.

## Introduction

In recent years a great variety of scholarly efforts have been directed at the development of efficiency measures. These measures illustrate whether the decision making units (DMUs) are near the production frontier. Farrell [1] seemed to be the first author who devoted his work to the study of the production frontier for evaluating productivity. A few years later, Farrell’s approach was developed to two major branches, including parametric estimation method and non-parametric estimation method. Moreover, data envelopment analysis (DEA), which is used to estimate the efficiency of the evaluated DMU relative to peer DMUs, is the basic non-parametric estimation method. Since then, many improved approaches on DEA have been proposed [2–16].

What we focus on in this paper is the DEA with a single output. The initial DEA model (CCR model), as originally presented in [17], was built on the earlier work of Farrell. This model allowed every DMU to select the most favorable weight while requiring the resulted ratios of weighted outputs to weighted inputs of all the DMUs to be not greater than 1. The CCR model is a fractional programming model and solved by transforming to a linear programming model. If the constraint $\sum^{n}_{j=1}\lambda_{j}=1$ is adjoined to the dual CCR model, the extended model is known as BCC model [18]. Soon afterwards, Charnes *et al.* [19] presented the C^2^GS^2^ model to establish foundations of DEA for Pareto-Koopmans efficient empirical production functions. Färe and Grosskopf introduced a non-parametric dual method (namely, FG model) to calculate the scale efficiency [20]. In 1990, Seiford and Thrall [21], who proposed the ST model, discussed the mathematical programming approach to frontier estimation, and examined the effect of model orientation on the efficient frontier and the effect of convexity requirements on returns to scale. In addition, the transformations between models were provided.

Unlike the previous models, we develop an extended evaluation approach to estimate the efficiencies of DMUs with a single output. Different from DEA models, the proposed approach will estimate the efficiencies of DMUs only with changed input data rather than with the original input and output data. Moreover, efficiency of the evaluated DMU is estimated by considering the relationship between the defined production possibility set and the corresponding line segment of the evaluated DMU. Furthermore, the minimal element is introduced in the paper to distinguish the weak efficiency and efficiency.

The rest of the paper is unfolded as follows. The initial DEA model, partially ordered set and minimal element are reviewed in Section 2. In Section 3, the extended evaluation approach for DMUs with a single output is proposed. In Section 4, an example is given to illustrate the presented approach. In Section 5, results and discussion are given. The paper is concluded in Section 6.

## An introduction to DEA model and the partially ordered set

As an extremely common DEA model, the CCR model assumes that there are *n* DMUs, and each DMU consumes the same type of inputs and produces the same type of outputs. Let *m*, *r* be the numbers of inputs and outputs, respectively. All inputs and outputs are assumed to be nonnegative, and at least one input and one output are positive. The multiple inputs and outputs of each DMU are aggregated into a single virtual input and a single virtual output. The efficiency of the evaluated DMU is obtained as a ratio of its virtual output to its virtual input, and is subject to the condition that the ratio for each DMU is not greater than 1. The corresponding model is as follows:

1 $$ (\mathit{CCR}) \textstyle\begin{cases} \max \frac{u^{T}y_{0}}{v^{T}x_{0}}\\ \text{s.t.}\\ \quad\frac{u^{T}y_{j}}{v^{T}x_{j}}\leq1,\quad j=1,\ldots, j_{0},\ldots,n,\\ \quad u\geq0,\qquad u\neq0,\\ \quad v\geq0,\qquad v\neq0, \end{cases} $$

where $x_{j}=(x_{j1}, \ldots, x_{jm})^{T}$ and $y_{j}=(y_{j1}, \ldots, y_{jr})^{T}$ are the input and output vectors of the *j*th DMU, and $j_{0}$ is the DMU under evaluation (usually denoted by DMU_0_). *u* and *v* are the weight column vectors of output and input, respectively. $u\geq0$, $u\neq0$ represents the vector whose elements are not less than zero but at least one element is a positive value. By applying the Charnes-Cooper transformation [22] in the model (1), the following equivalent linear model is obtained:

2 $$ (P_{\mathit{CCR}}) \textstyle\begin{cases} \max \mu^{T}{y_{0}}\\ \text{s.t.}\\ \omega^{T}x_{j}-\mu^{T}y_{j}\geq0,\quad j=1,\ldots,n,\\ \quad\omega^{T}x_{0}=1,\\ \quad\omega\geq0, \qquad\omega\neq0,\\ \quad\mu\geq0, \qquad\mu\neq0. \end{cases} $$

The essence of the model above is to find the weight vector to maximize its weighted output of the evaluated DMU, and the weighted output is not greater than the weighted input for every DMU. Moreover, the optimal objective values of DEA models vary in $(0, 1]$. The relationship between DEA efficiency and optimal objective value can be obtained as follows.

### Definition 1

If the optimal objective value of the evaluated DMU is equal to 1 and there is at least one optimal solution in which the optimal weight vectors of inputs and outputs are greater than 0, then the evaluated DMU is DEA efficient.

### Definition 2

If the optimal objective value of the evaluated DMU is equal to 1 and there is not any optimal solution in which the optimal weight vectors of inputs and outputs are greater than 0, then the evaluated DMU is weak DEA efficient.

### Definition 3

If the optimal objective value of the evaluated DMU is less than 1, then the evaluated DMU is DEA inefficient.

Subsequently, the partially ordered set and minimal element will be introduced. Let *P* be a nonempty set. Any subset of the cartesian product set $P\times P=\{(x,y)|x,y\in P\}$ is called a binary relation, denoted by *R*. $a,b\in P$, $aRb$ if and only if $(a, b)\in R$ [23].

### Definition 4

A relation R is called a partial order on P if it satisfies, for all $x,y,z\in P$,

1. reflexivity, $x R x$,
2. antisymmetry, $x R y$ and $y R x$ imply $x=y$,
3. transitivity, $x R y$ and $y R z$ imply $x R z$.

A nonempty set *P* equipped with a partial order is called a partially ordered set, or poset for short. A partial order *R* is traditionally replaced by ‘≤’. That is, we usually replace $x R y$ by $x \leq y$ which is read as ‘*x* is less than or equal to *y*’.

### Definition 5

Suppose that *P* is a partially ordered set and $Q\subseteq P$, $a\in Q$ is called a minimal element of *Q* if $a\geq x$ and $x\in Q$ imply $a=x$.

For any nonempty finite subset $S\subseteq P$, there exists at least one minimal element $x\in S$.

## The extended evaluation approach

In this section, a new evaluation approach for DMUs with a single output is proposed by considering the relationship between the defined production possibility set and the line segment joining the origin to the evaluated DMU.

It should be noted that all DMUs used from this section onwards are in the form of a single output. Now suppose there are *n* DMUs with *m* inputs and one output. Especially, $X_{k}=\{x_{k1}, \ldots, x_{km}\}$ denotes the input vector of the *k*th DMU with *k* ranging from 1 to *n*. Without loss of generality, $Y_{k}=\{y_{k}\}$ is the output vector with a single element for the *k*th DMU. Since efficiency is independent of the changes of inputs and output by the same proportion, then we change the input and output data of each DMU in the same proportion until the output data of all DMUs are equal. The input vector and output vector of the *k*th DMU are transformed into $\overline{X}_{k}=\{\bar{x}_{k1}, \ldots, \bar{x}_{km}\}$ and $\overline{Y}_{k}=\{\bar{y}_{k}\}=\overline{Y}_{l}$, $l=1, \ldots, n$. In order to discuss the convenience of the problem, the output state will not be considered in the evaluation approach.

Next, we define the production possibility set $T_{i}$ which is spanned by all the DMUs without the *i*th evaluated DMU. For instance, if there are five DMUs (*i.e.*, DMU_1_, DMU_2_, DMU_3_, DMU_4_, and DMU_5_), and DMU_3_ is the evaluated DMU, then the production possibility set $T_{3}$ is spanned by DMU_1_, DMU_2_, DMU_4_, and DMU_5_. Moreover, the production possibility set $T_{i}$ satisfies the following conditions:

1. $\overline{X}_{k}\in T_{i}$, $k\neq i$.
2. For arbitrary $\overline{X}_{k}, \overline{X}_{l}\in T_{i}$, and $\alpha\in[0, 1]$, we have $\alpha\overline{X}_{k}+ (1-\alpha)\overline {X}_{l}\in T_{i}$.
3. If $\overline{X}_{k}\in T_{i}$, and $\overline{X}_{l}\geq\overline {X}_{k}$, then $\overline{X}_{l}\in T_{i}$.
4. If $\overline{X}_{k}\in T_{i}$, and $\alpha\geq1$, then $\alpha \overline{X}_{k}\in T_{i}$.
5. $T_{i}$ is the least set which satisfies the conditions (1)–(4).

The production possibility set $T_{i}$ spanned by $\overline{X}_{k}=\{ \bar{x}_{k1}, \ldots, \bar{x}_{km}\}$, $k \neq i$ is given by the following formula:

3 $$ T_{i}= \Biggl\{ X \Big| \sum^{n}_{k=1, k\neq i} \lambda_{k}\overline {X}_{k}\leq X, \lambda_{k} \geq0, \sum^{n}_{k=1, k\neq i}\lambda_{k}=1 \Biggr\} , \quad i=1, \ldots, n. $$

### The relationship between the defined production possibility set and the line segment for the evaluated DMU

To better show the proposed approach, we will consider the case with two inputs and one output. Then we change the inputs and output of each DMU in the same proportion until output data of all the DMUs are equal. Next, the coordinate system is established with input 1 and input 2 as the *x* and *y* coordinate axes. For the DMU under evaluation, the closer it gets to the coordinate origin, the higher production efficiency will be.

**Figure 1:**
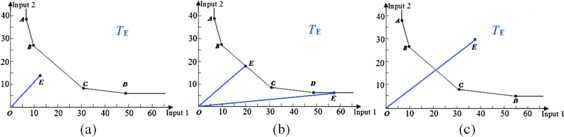
**Efficiency analysis of the evaluated DMU.**

In this section, there are five DMUs (*i.e.*, DMUs *A*, *B*, *C*, *D* and *E*) with the same output data, and DMU *E* is the evaluated DMU, then the production possibility set is spanned by DMUs *A*, *B*, *C* and *D*. As shown in Figure 1, the solid line segments connecting points *A*, *B*, *C* and *D* constitute an isoquant that represents the different input amounts to produce the same output amount. Since it is impossible to reduce the amount of one of the inputs without increasing another input amount if one is to stay on this isoquant, the solid line segments represent the efficient production frontier [24] of the production possibility set $T_{E}$.

From Figure 1(a) we can see that the evaluated DMU *E* is closer to the coordinate origin than the production frontier (that is to say, the line segment *OE* and the production possibility set $T_{E}$ are disjoint). There exist the optimal weight vectors of inputs and output such that the production efficiency of DMU *E* is higher than DMUs *A*, *B*, *C* and *D*, then DMU *E* is efficient for DMUs *A*, *B*, *C*, *D* and *E*. In other words, the evaluated DMU *E* is efficient if there is no solution of the inequalities of the production possibility set $T_{E}$ and the line segment *OE*.

In Figure 1(b), the evaluated DMU *E* is located on the production frontier (namely, the line segment *OE* meets on the production possibility set $T_{E}$ at a point *E*, and there is exactly one solution of the inequalities of the production possibility set $T_{E}$ and the line segment *OE*). There are two cases of interest: (1) The evaluated DMUs *E* on the three solid line segments *AB*, *BC* and *CD* are efficient. (2) The evaluated DMUs *E* on the two rays issuing from the points *A* and *D* are weakly efficient [25, 26]. It is important to stress here that at least one input of the weakly efficient DMU is strictly greater than that of an efficient DMU. Moreover, if the order relation ≤ for DMUs *A*, *B*, *C*, *D* and *E* is reflective, antisymmetric and transitive in the coordinate system, then the set $\{ A, B, C, D, E\}$ is a partially ordered set. In the proposed approach, with the same output for all DMUs, if an evaluated DMU is located on the production frontier, its efficiency is dependent on whether the evaluated DMU is a minimal element or not. An evaluated DMU on the production frontier is weakly efficient if it is not a minimal element, otherwise the evaluated DMU is efficient.

Refer to Figures 1(c), since DMU *E* is farther from the coordinate origin than the production frontier, that is, the line segment *OE* meets on the production possibility set $T_{E}$ at more than one point, DMU *E* is located in the defined production possibility set $T_{E}$, thus DMU *E* is inefficient. In such a case, the solution of inequalities of the production possibility set and the line segment is not unique.

From the analysis mentioned above, the conclusion thus noted may be recorded as

#### Theorem 1

*Suppose there are* *n* *DMUs* (*i*.*e*., *DMU* $E_{1}, \ldots, E_{n}$) *with* *m* *inputs and one output*, *and output data of all DMUs are equal*. *Then an* *m*-*dimensional coordinate system is established with input* *i* *as the* *ith coordinate axis*, *the point* $E_{k}$ *stands for the* *kth DMU in the coordinate system*, *and* $OE_{k}$ *denotes the line segment from the origin to* $E_{k}$. $T_{k}$, *spanned by other* $n-1$ *DMUs*, *is the production possibility set of the* *kth DMU*. *Efficiency is obtained from the relationship between line segment* $OE_{k}$ *and the production possibility set* $T_{k}$ *for the* *kth evaluated DMU is as follows*:

1. *If the line segment* $OE_{k}$ *and the production possibility set* $T_{k}$ *are disjoint*, *that is*, *there is no solution of the inequalities of the production possibility set* $T_{k}$ *and the line segment* $OE_{k}$, *then the* *kth DMU is efficient*.
2. *The line segment* $OE_{k}$ *meets on the production possibility set* $T_{k}$ *at the point* $E_{k}$, *which is located on the production frontier*, *that is*, *there is exactly one solution of the inequalities of the production possibility set* $T_{k}$ *and the line segment* $OE_{k}$. *If the evaluated DMU* $E_{k}$ *is not a minimal element of* $\{E_{1}, \ldots, E_{n}\}$ *equipped with an order relation* ≤, *then DMU* $E_{k}$ *is weakly efficient*, *otherwise DMU* $E_{k}$ *is efficient*.
3. *If the line segment* $OE_{k}$ *meets on the production possibility set* $T_{k}$, *and the number of points of intersection are greater than one*, *that is*, *the number of solutions of the inequalities of the production possibility set* $T_{k}$ *and the line segment* $OE_{k}$ *is greater than one*, *then the* *kth DMU is inefficient*, *and* $E_{k}$ *is located in the production possibility set*.

Notice that the number of points of intersection of $OE_{k}$ and $T_{k}$ is equal to the number of solutions of equation (3) and the expressions of line segment $OE_{k}$. If the input vector of DMU $E_{k}$ is $\overline{X}_{k}=\{\bar{x}_{k1}, \ldots, \bar{x}_{km}\}$, the expressions of line segment $OE_{k}$ are as follows:

$$\textstyle\begin{cases} \bar{x}_{1}=\lambda\bar{x}_{k1}, \\ \bar{x}_{2}=\lambda\bar{x}_{k2}, \\ \vdots\\ \bar{x}_{m}=\lambda\bar{x}_{km}, \\ \lambda\in(0, 1]. \end{cases} $$

## Numerical experiments

In this section, an example is given to illustrate the practical relevance of the presented approach. In the example, there are five DMUs (*i.e.*, DMUs *A*, *B*, *C*, *D* and *E*) with two inputs and a single output listed in Table 1.

**Table 1:** **DMUs with two inputs and a single output**

***DMU***	***A***	***B***	***C***	***D***	***E***
*Input 1*	2	3	9	1	4
*Input 2*	6	3	3	6	4
*Output*	2	0.5	3	1	2

At first, we change the inputs and output of every DMU in the same proportion until the output data are equal to 1, and establish the coordinate system with input 1 and input 2 as the *x* and *y* coordinate axes. The dots (•) denote the corresponding DMUs with the same output.

**Figure 2:**
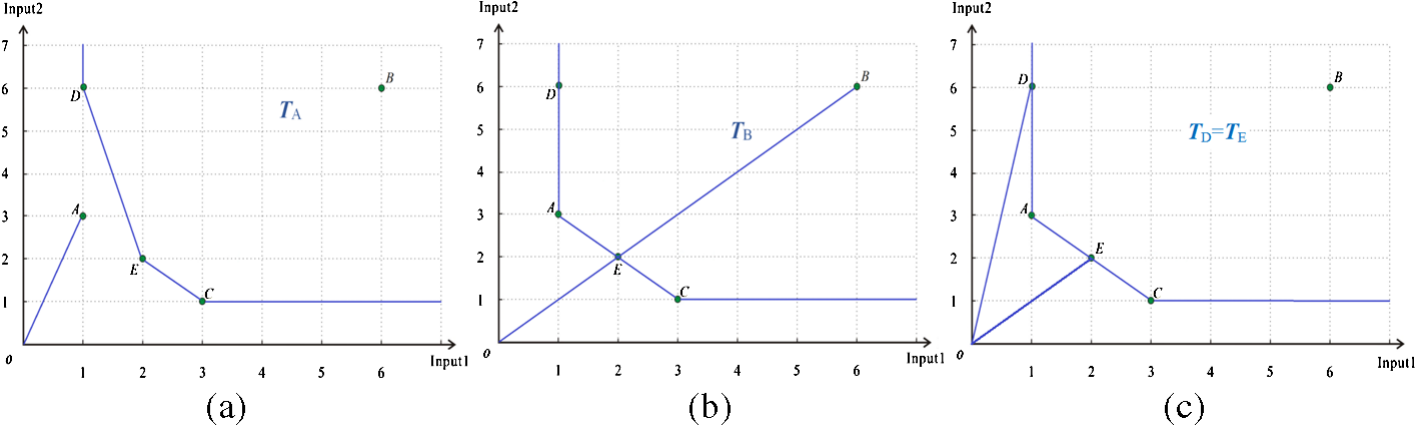
**Efficiency analysis of DMUs** ***A*** **,** ***B*** **,** ***D*** **and** ***E*** **.**

Next, the DMU *A* will be estimated, and the evaluation process is as follows. The production possibility set $T_{A}$ is spanned by DMUs *B*, *C*, *D* and *E* (see Figure 2(a)). By using equation (3), we get the following expression:

$$T_{A}= \Biggl\{ X \Big| \sum^{5}_{k=2} \lambda_{k}\overline{X}_{k}\leq X, \sum ^{5}_{k=2}\lambda_{k}=1, \lambda_{k}\geq0, k=2,3,4,5 \Biggr\} , $$

that is,

4 $$ \textstyle\begin{cases} 6\lambda_{2}+3\lambda_{3}+\lambda_{4}+2\lambda_{5}\leq\bar{x}_{1}, \\ 6\lambda_{2}+\lambda_{3}+6\lambda_{4}+2\lambda_{5}\leq\bar{x}_{2}, \\ \lambda_{2}+\lambda_{3}+\lambda_{4}+\lambda_{5}=1, \\ \lambda_{k}\geq0,\quad k=2, 3, 4, 5. \end{cases} $$

In addition, the expressions of line segment *OA* are

5 $$ \textstyle\begin{cases} \bar{x}_{1}=\lambda, \\ \bar{x}_{2}=3\lambda, \\ \lambda\in(0, 1]. \end{cases} $$

The relationship between the line segments *OA* and the production possibility set $T_{A}$ for the evaluated DMU *A* will be analyzed. As shown in Figure 2(a), the line segments *OA* and the production possibility set $T_{A}$ are disjoint, and there is no solution for inequalities (4) and (5). By applying Theorem 1, we conclude that DMU *A* is efficient.

6 $$ \textstyle\begin{cases} \lambda_{1}+3\lambda_{3}+\lambda_{4}+2\lambda_{5}\leq\bar{x}_{1}, \\ 3\lambda_{1}+\lambda_{3}+6\lambda_{4}+2\lambda_{5}\leq\bar{x}_{2}, \\ \lambda_{1}+\lambda_{3}+\lambda_{4}+\lambda_{5}=1, \\ \lambda_{k}\geq0, \quad k=1, 3, 4, 5,\\ 6\lambda=\bar{x}_{1}, \\ 6\lambda=\bar{x}_{2}, \\ \lambda\in(0, 1]. \end{cases} $$

Similarly, the production possibility set $T_{B}$ of the evaluated DMU *B* is shown in Figure 2(b), and the inequalities of the production possibility set $T_{B}$ and line segment *OB* are expressed by (6). In Figure 2(b), we see that DMU *B* is located in the production possibility set $T_{B}$, while there are infinite solutions for (6), so DMU *B* is inefficient.

7 $$ \textstyle\begin{cases} \lambda_{1}+6\lambda_{2}+3\lambda_{3}+2\lambda_{5}\leq\bar{x}_{1}, \\ 3\lambda_{1}+6\lambda_{2}+\lambda_{3}+2\lambda_{5}\leq\bar{x}_{2}, \\ \lambda_{1}+\lambda_{2}+\lambda_{3}+\lambda_{5}=1, \\ \lambda_{k}\geq0,\quad k=1, 2, 3, 5,\\ \lambda=\bar{x}_{1}, \\ 6\lambda=\bar{x}_{2}, \\ \lambda\in(0, 1]. \end{cases} $$

The same spanning production possibility sets of DMUs *D* and *E* are shown in Figure 2(c), the inequalities of DMUs *D* and *E* are expressed by (7) and (8) separately. The two line segments *OD* and *OE* meet on the production possibility set $T_{D}=T_{E}$ at the production frontier, and there is only one solution for (7) and (8), respectively. In addition, the data of Input 1 of DMUs *D* and *A* are equal, but the data of Input 2 of DMU *D* are greater than those of DMU *A*, and DMU *D* is not a minimal element of $\{A, B, C, D, E\}$ with the order relation ≤, so DMU *D* is weakly efficient. DMU *E* is a minimal element of partially ordered set $\{A, B, C, D, E\}$, so it is efficient. Moreover, DMU *E* can be expressed by a linear combination of DMU *A* and *C*.

8 $$ \textstyle\begin{cases} \lambda_{1}+6\lambda_{2}+3\lambda_{3}+\lambda_{4}\leq\bar{x}_{1}, \\ 3\lambda_{1}+6\lambda_{2}+\lambda_{3}+6\lambda_{4}\leq\bar{x}_{2}, \\ \lambda_{1}+\lambda_{2}+\lambda_{3}+\lambda_{4}=1, \\ \lambda_{k}\geq0,\quad k=1, 2, 3, 4,\\ 2\lambda=\bar{x}_{1}, \\ 2\lambda=\bar{x}_{2}, \\ \lambda\in(0, 1]. \end{cases} $$

At last, by a similar evaluation process, we consider the DMU *C*, and the result is shown in Table 2. We can see that the evaluation results are consistent with the results from model (2).

**Table 2:** **The efficiencies of DMUs**

***DMU***	***A***	***B***	***C***	***D***	***E***
*DEA efficiency of model (* *2* *)*	*DEA efficient*	*DEA inefficient*	*DEA efficient*	*Weak DEA efficient*	*DEA efficient*
*Efficiency of the proposed approach*	*Efficient*	*Inefficient*	*Efficient*	*Weak efficient*	*Efficient*

## Results and discussion

The main purpose of this paper is to develop a new evaluation approach to estimate the efficiencies of DMUs with *m* inputs and a single output. The efficiency measure is independent of the changes of inputs and outputs by the same proportion for the DMUs. In such a case, we change the inputs and output of each DMU in the same proportion until all the output data are equal, and then establish an *m*-dimensional coordinate system with input *i* as the *i*th coordinate axis. Subsequently, in the coordinate system, the production possibility set, which is spanned by all the DMUs except the DMU under evaluation, is defined by a formula that can be prompted by inequalities. In addition, the line segment joining the origin to the evaluated DMU is employed in the proposed approach, and its expression is given. Efficiency of the evaluated DMU is estimated by the relationship between the production possibility set and the line segment. Generally speaking, for the production possibility set and the line segment of each DMU, there are three intersecting results which can be obtained from the graphical method or the analytical method. In order to determine the efficiency of the evaluated DMU, the theorem is established to elucidate the relationship between the intersecting results and the efficiencies. Moreover, the partially ordered set and minimal element are used to distinguish the weak efficiency and efficiency. Finally, the use of the proposed approach is illustrated by means of an example.

## Conclusions

The results of the proposed approach are consistent with the results of the DEA model. It is worthy of note that the inequality approach can also be applied to super-efficiency DEA. If there is no solution for the inequalities, the evaluated DMU is super-efficient. If the solution of inequalities is not unique, the evaluated DMU is inefficient. If there is exactly one solution, and the evaluated DMU is a minimal element of all the DMUs, then the evaluated DMU is efficient, otherwise the evaluated DMU is weakly efficient.
